# Caring for caregivers and persons living with dementia under home-based primary care: protocol for an interventional clinical trial

**DOI:** 10.1186/s40814-024-01455-x

**Published:** 2024-02-09

**Authors:** Maimouna Sy, Ayush Thacker, Orla C. Sheehan, Bruce Leff, Christine Seel Ritchie

**Affiliations:** 1 Center for Aging and Serious Illness, Department of Palliative Care and Geriatric Medicine, Massachusetts General Hospital, Boston, MA USA; 2 Johns Hopkins University School of Medicine, Baltimore, MD USA; 3 Royal College of Surgeons in Ireland, Dublin, Ireland

**Keywords:** Dementia, Home-based primary care, Caregiver, Intervention, Dementia care

## Abstract

**Background:**

Approximately 7.5 million older adults are homebound, who have difficulty and/or need assistance to leave their homes. In this growing population, the prevalence of people living with dementia (PLWD) is approximately 50%. Current dementia care models in the USA were developed for traditional office-based primary care and have not been tailored to home-based primary care (HBPC) delivery models. Literature has shown that office-based collaborative interventions can improve caregiver outcomes including caregiver stress, well-being, and morbidity and patient outcomes including improved quality of life and reduced emergency department visits (Possin KL, Merrilees JJ, Dulaney S, Bonasera SJ, Chiong W, Lee K, JAMA Int Med 179:1658, 2019). To date, the evidence for HBPC dementia interventions is lacking. Though HBPC has demonstrated benefit in homebound older adults, there is limited literature on the effects of HBPC on persons living with dementia (Nguyen HQ, Vallejo JD, Macias M, Shiffman MG, Rosen R, Mowry V, J Am Geriatr Soc 70:1136–46, 2021). Our goal is to develop a HBPC-focused dementia care intervention that integrates the components of two previously developed dementia care models and test the feasibility of implementing it in HBPC practices to improve the quality of life and wellbeing of homebound PLWD and their caregivers.

**Methods:**

We will first conduct qualitative focus groups at two HBPC practice sites, one in the Southeast and one in Hawaii in order to obtain preliminary feedback on the proposed intervention. At each site, there will be one focus group with caregivers of PLWD and another with HBPC clinicians and staff to help develop and refine our intervention. We will then conduct an open-pilot trial of the refined intervention at the two HBPC practices. A total of up to 25 patient/caregiver dyads will be recruited at each site (*N* = 50 total). Outcomes measured through pre-and-post assessments and exit interviews will include (a) feasibility for the caregiver to engage with and complete baseline assessments and access educational materials and community resources and (b) feasibility for the practice to identify potential caregivers/patients, assess eligible patient/caregiver dyads, use patient and caregiver assessments, recruit patient/caregiver dyads, recruit racial and ethnic minorities, use care modules, and engage with the tele-video case conference, (c) net promoter score, (d) acceptability of the intervention to caregivers and patients to participate in the intervention, (e) caregivers feeling heard and understood, and (f) caregiver well-being.

**Discussion:**

Testing the feasibility and acceptability of the adapted intervention in these two HBPC practices will provide the basis for future testing and evaluation of a fully powered intervention for PLWD and their caregivers cared for in HBPC with the goal of disseminating high-quality and comprehensive dementia-care focused interventions into HBPC practices.

**Trial registration:**

This trial was registered with ClinicalTrials.gov NCT05849259 in May 2023.

## Introduction

### Background

Approximately 7.5 million older adults in the USA are mostly homebound, who have difficulty and/or need assistance to leave their homes [1] due to the combined effects of substantial chronic disease burden and functional impairments [2]. During the COVID-19 pandemic, homebound prevalence more than doubled to nearly 13% with greater rates of increase among racial and ethnic minorities [3].

Traditional office-based primary care is often inaccessible to the homebound due to their functional limitations. Home-based primary care (HBPC) provides longitudinal medical care in the patient’s home [4], usually by a team of doctors, nurse practitioners, nurses, physician assistants, social workers, and other clinical personnel. HBPC aims to improve the quality of life and health outcomes of patients and their families, while reducing the cost of care [5]. This model of care has been shown to reduce hospital admissions, emergency department visits, long-term care admissions, and the duration of hospital stays [6].

Dementia is highly prevalent among homebound older adults (between 40 and 80%) and is associated with high morbidity and mortality [1]. Caregivers of people living with dementia (PLWD) provide critical support and commonly suffer from significant stress, morbidity, and mortality due to their caregiving role [1] including depression, anxiety, and low levels of emotional well-being [7]. Given the lack of access to traditional primary care for homebound older adults living with dementia, it is vital that HBPC practices have the tools to provide high-quality care to address the needs of PLWD and their caregivers.

Dementia-specific care models have been developed for traditional office-based co-management with primary care, as stand-alone entities, and as part of population health programs [8]. Care Ecosystem [9], an existing dementia care model, uses a dedicated health care navigator trained to implement three core components to improve outcomes for PLWD and their caregivers: (1) standardized assessments, (2) standardized evidence-based interventions based on findings from those assessments, and (3) a team-based review and case-conference to collaborate on ideas for solving care challenges experienced by participants [9]. Care Ecosystem demonstrated improved patient quality of life, reduced emergency department visits, reduced caregiver burden and depression levels, and increased competency in office-based clinicians for the unmet needs in persons living with dementia and their caregivers [9]. CaRe Ecosystem primary Care Embedded demeNtia Treatment (CRESCENT) was adapted from Care Ecosystem to allow for improved integration with care managers associated with an office-based primary care setting [10]. This model retained the three core components but used office-based clinicians instead of dedicated healthcare navigators to conduct the intervention.

Because HBPC is inherently different from traditional office-based primary care, interventions such as Care Ecosystem and CRESCENT may need to be adapted to be suitable for primary care in the home setting. HBPC practices do not commonly have assets such as health care navigators and the workflows of HBPC are significantly different from traditional office-based primary care. Developing a dementia care intervention appropriate for HBPC practices that is centered on the person living with dementia and their caregiver as a unit may improve outcomes.

## Project overview

The objective of this study is to develop a new intervention, Dementia Care Quality at Home, by adapting the Care Ecosystem and CRESCENT models to HBPC, and to test the feasibility, acceptability, and fidelity of its implementation in the HBPC context. This new model will be adapted from Care Ecosystem and CRESCENT with substantial input from HBPC clinicians and staff to tailor the intervention appropriately to HBPC.

The draft model will adapt the three core components used in Care Ecosystem and CRESCENT: standardized assessments, use of evidence-based interventions based on findings from those assessments, and a team-based review and case-conference. Designated dementia champion(s) at each practice (clinicians and staff) will implement the intervention. The baseline assessment will include direct observation of the home environment and the interpersonal dynamics between PLWD and their caregivers to help develop the care plan. The standardized evidence-based interventions will be adapted for the home and the unique clinical workflow of HBPC. The team-based case conference will be adapted to include both practice clinicians and national dementia experts with HBPC dementia care experience to problem-solve clinical challenges and promote continual learning. This format will reflect that used by Project Extension for Community Healthcare Outcomes, which was constructed to promote the collaboration between primary care providers (“frontline clinicians”) and medical specialists to address individual challenges and exchange best practices to overall increase dementia knowledge, expertise, and self-efficacy in home-based primary care practices [11].

After adapting the intervention, we will conduct an open-pilot of the HBPC-tailored intervention at two culturally diverse HBPC practices in Honolulu, Hawaii, and in Richmond, Virginia. The HBPC practice in Hawaii is 68% Asian, 19% White, and 10% Native Hawaiian/Pacific Islander. The population of the HBPC practice in Virginia is 55% Black or African American, 36% White, and 8% Other. The inclusion of practices with diverse patient populations is especially important given the disparities already evident in the homebound population [3].

## Methods

### Study design

The first aim is to develop and refine the intervention. This will be accomplished by conducting qualitative focus groups with caregivers of PLWD and HBPC clinicians and staff to obtain real-world input to tailor the intervention to the unique needs of both homebound PLWD and their caregivers and the HBPC practices that care for them. The second aim will involve training HBPC clinicians and staff in the two different HBPC practices to implement the intervention in an open pilot to determine the feasibility, acceptability, and fidelity in implementing the Dementia Quality Care at Home intervention. The trial is registered in ClinicalTrials.gov NCT05849259.

### Overview

The study was approved by the Massachusetts General Brigham Institutional Review Board in January 2023 and received single Institutional Review Board approval in February 2023. In quarters 1 and 2, the user-centered adaptation will be conducted. During this adaptation, there will be patient and caregiver recruitment and baseline assessments of the HBPC practices, patients, and caregivers. In quarters 3 and 4, the open pilot will be conducted followed by follow-up surveys of practices and patients in quarters 5 and 6. In quarter 6, there will be initial manuscript preparation, scientific talks, and preparation for a larger multi-site efficacy trial.

### Phase 1: Focus groups

In phase 1, the study team will conduct qualitative focus groups with caregivers of PLWD and HBPC clinicians and staff to obtain input on how to best refine and adapt features of Care Ecosystem and CRESCENT into the Dementia Care Quality at Home intervention. The team will engage in purposive sampling to assure representation of historically minoritized groups. Sites will be asked to identify caregivers from diverse backgrounds. While sites will enroll participants based on inclusion criteria, they will also be asked to choose dyads who fully represent the demographic composition of their community. Each site will hold two focus groups, one for caregivers of PLWD and the other for HBPC clinicians and staff, with up to twelve people per group. Focus groups will last up to one hour and will be audio-recorded and transcribed using Zoom, a video communications platform. A trained research member will facilitate the discussion, ask individuals to describe their lived experience with dementia, and provide feedback on the proposed intervention. Two members of the research team will independently review the focus group transcripts and identify relevant themes and sub-themes. The research team will approach the analysis through the Framework Method. This method includes qualitative thematic analysis with a hybrid deductive-inductive analysis plan. In this open pilot phase, we focus on feasibility benchmarks and not on evidence of efficacy. All data will be de-identified and participants will be assigned an identification number.

### Population

Patients and caregivers will be identified through a screening process by the HBPC clinicians. HBPC clinicians will identify all patients with a dementia diagnosis and, consequently, consider them for the screening process based on the availability of a caregiver and their needing help with at least 2 instrumental activities of daily living or 1 activity of daily living. In addition, eligible patients will need to exhibit at least one dementia-related behavioral abnormality: delusions, hallucinations, agitation/aggression, depression/dysphoria, anxiety, elation/euphoria, apathy/indifference, disinhibition, irritability/lability, motor disturbances, irregular nighttime behaviors, and/or disruptions in appetite/eating. Patients with all severities of dementia will be eligible for recruitment; however, if behavioral symptoms are caused by other serious mental illnesses such as schizophrenia or bipolar disorder, patients are ineligible for enrollment. Likewise, those receiving hospice care at the time of enrollment will be ineligible. Patients will not become ineligible if they start receiving hospice care after enrollment. In addition, caregivers must experience caregiver-related distress in their caregiver role (must be over 18 years or older), have English fluency and literacy, live in the USA, live with and care for an individual with Alzheimer’s disease and Alzheimer’s disease-related dementias (ADRD), anticipate providing care for the next 6 months, and provide an average 4 h of supervision or direct assistance per day for the care recipient.

Clinicians will be identified as practice staff members that are a part of a HBPC primary program or closely connected to the practice, are 18 years or older, live in the USA, and have English fluency and literacy. The HBPC sites in both Hawaii and Virginia represent diverse clinical practices with staff of different racial and ethnic backgrounds to provide concordant care to their patient populations.

### Focus group domains

The key domains of the HBPC clinician and staff focus groups will include the following: (1) barriers and facilitators to implementation of the intervention into HBPC practices, (2) adaptations to the protocol to increase relevance in the home care setting and to encourage caregiver engagement and retention, (3) barriers and facilitators in using clinical staff to deliver the intervention, (4) adaptations to the content to best accommodate the setting and population, and (5) barriers the HBPC practice may experience in delivering the intervention. The key domains of the caregivers of PLWD focus groups will include the following: (1) caregiver support, (2) community resources for PLWD, (3) medication management, (4) decision-making and future planning around care settings, treatments, finances, and care, (5) managing challenging behaviors including agitation, aggression, physical violence, sundowning and sleep problems, and (6) managing safety issues such as falls, wandering out of the home, and using tools or kitchen appliances.

### Phase 2: Open-pilot trial

The open pilot trial will assess the feasibility, acceptability, and fidelity of the Dementia Care Quality at Home intervention at two HBPC practices. Outcomes will be measured through pre- and post-assessments. Each site will recruit up to 25 patient/caregiver dyads, totaling 50 patient/caregiver dyads across both sites. Through this open-pilot trial, HBPC practice dementia champions will receive training and then implement the intervention into routine care. Outcome measures are delineated in Table 1.

**Table 1:** Outcome measures

**Domain**	**Measure**	**Instrument**	**Data source**
Engage with and complete baseline assessments	> 70% of caregivers (CGs) will complete baseline assessments	Baseline assessments	Practice staff
Feasibility to access educational materials and community resources	> 70% of CGs will report using 1 or more materials provided by the practice	Caregiver survey	Study team
Any participation in the intervention	% of CGs invited to participate who agree to participate in the intervention	Practice log	Practice
Satisfaction with the intervention	% of CGs who agree or strongly agree that they were satisfied with the intervention	Caregiver survey	Research team
Net promoter score	% of CGs who report that they felt heard and understood by the practice % of CGs who report that the intervention helped with care of their loved one % of CGs who report that they would recommend the intervention to another CG	Caregiver survey	Research team
Caregiver well-being	Quality of Life in Alzheimer’s Disease	Survey	Research team
Identification of potential patients/CG participants in intervention	Ability of practice to generate list of their patients living with dementia	Log	Practice electronic health record (EHR)
Assessment of eligibility pt/CG dyads for intervention	Ability of practice to identify eligible patient/caregiver dyads (e.g., CG experiencing burden or distress)	Log	Practice EHR
Recruitment of patient and CG (overall, and % of racial and ethnic minorities)	> 50% of eligible dyads will enroll > 35% of recruited dyads are racial and ethnic minorities (by practice)	Log	Practice log
Use of patient and CG assessments	> 75% practice personnel who conduct assessments will rate assessments as feasible to use, and > 75% of audited assessments will be completed	Survey of practice personnel conducting assessments Practice EHR audits of assessments	Survey of practice clinicians conducting assessments Practice EHR audits of assessments
Use of care modules	> 75% will rate modules as feasible to use, > 75% practice personnel who complete the modules will rate the modules as feasible to use, and > 50% of audited modules will be completed	Survey	Survey of practice clinicians and staff
Clinicians to engage with the tele-video case conference	> 75% of virtual meetings attended	Attendance log	Zoom logs

### Population

Patients, caregivers, and practice staff will be identified through a similar process as outlined in phase 1.

### Intervention

The Dementia Care Quality at Home intervention training will utilize components from Care Ecosystem and CRESCENT: a standardized assessment tool used to determine the needs of the person living with dementia and their caregiver, six modules aimed to increase the well-being of the person living with dementia and their caregiver, and a team-based review and case-conference to review and collaborate on ideas to solve care challenges experienced by participants. The six planned modules are as follows: medication reconciliation and review, safety screening, community resources and caregiver education, caregiver well-being, behavior management, and decision making and advance care planning. It will incorporate an asynchronous online dementia training with quizzes on the six modules and synchronous training which will address the following: the assessment and follow-up modules (experiential training), communication skills, documentation expectations and workflow, and tele-video clinical case conferences to address questions and challenges that arise (reflective practice). The dementia champions at the practice will undergo all training and be responsible for conducting the intervention at their practice. This training is standardized for all clinicians who will be providing interventional care to ensure a consistent approach to enrolling and providing services to patients and caregivers. They will reach out to caregivers of persons living with dementia and caregivers will evaluate the intervention before and after receiving all six modules. By incorporating the principles of previous dementia care models and adapting the intervention as needed based on the practice’s individual needs and based on the population they serve, this study will facilitate an evidence-focused approach to providing high-quality dementia care and overall improving health outcomes in patients with dementia and their caregivers.

### Outcomes

Table 1 depicts outcome measures of the open pilot for caregivers of PLWD and for the HBPC practice. The feasibility of the intervention for caregivers will be assessed by rates of baseline assessment completion and the rate of use of educational materials and community resources by caregivers. Acceptability of the intervention for caregivers will be measured by their participation in all 3 components of the intervention. Satisfaction with the intervention for caregivers will be measured through surveys on how much the caregivers felt heard and understood by their practice, if they felt the intervention helped improve care for their loved one, and if they would recommend the intervention to another caregiver of a person living with dementia. Caregiver well-being will be measured through a post-intervention survey. Feasibility of the intervention for the practice will be measured via practice logs or electronic health records by identifying and assessing the proportion of eligible patient/dyads recruited for the intervention, caregiver recruitment, and, overall, how many participants reflected the racial and ethnic composition of the practice. Acceptability of the intervention for the practice will be measured through the rate at which clinicians were able to provide the intervention at their practice site and their own ratings of the acceptability of the intervention. The impact of the intervention on the practice will be measured through post-intervention focus groups with practice staff.

### Sample size

Consistent with guidelines for feasibility pilot studies, the primary focus of this trial is on feasibility of processes and procedures. We will use the approach by Lewis et al. to identify the sample size for this study using our feasibility, acceptability, and fidelity criteria thresholds [12]. Using normal approximation and a 1-sided *α* = 0.05, a sample size of *n* = 50 will have > 90% power to confirm a “go” criteria of 75% and a no-go criteria of 50%.

### Analysis plan

For focus groups, we will develop an a priori standardized coding scheme from our semi-structured interview guides. We will code transcripts using qualitative analysis software and assess agreement to ensure reliability of coding. We will use a hybrid deductive-inductive approach to analysis by using our standardized coding scheme and integrating new themes as they emerge in the coding process [13]. We will structure our inductive analyses based on the Framework Method [14].

We will calculate feasibility, acceptability, and fidelity benchmarks and present results as proportions with 95% confidence intervals.

## Discussion

### Expected findings

The expected outcomes of aim 1 are to learn from caregivers of PLWD on how a HBPC practice can better serve them in several key medical and social domains. This input will help optimize the design of the intervention to best meet their needs. From clinicians, the expected outcomes are to learn how to optimize the intervention and how to best implement the intervention into the HBPC practice workflow while being mindful of the diverse population the practice serves. The expected outcomes of aim 2 are to determine the feasibility and acceptability of the intervention to the practices and caregivers of PLWD. The objective is that by receiving feedback from focus groups and through testing out the intervention, an intervention can be developed and implemented that adequately supports HBPC practices and PLWD and their caregivers who receive HBPC. The data obtained from this study will contribute to the development and the implementation of the Dementia Care Quality at Home intervention, which can be tested in future clinical trials.

### Practical or operational issues

In performing a multi-site study with diverse practices, it is important to create a consistent line of communication among the team. This is best accomplished by holding weekly or biweekly meetings with the larger team to discuss study progress and troubleshoot any questions or challenges. As each site will have their own internal institutional review board (IRB) requirements, it is recommended that there is ample time budgeted for their submission and approval. Meetings with the sites’ IRB persons will also be helpful in ensuring that all requirements for each site are met. For sites who will serve as the “parent site,” i.e., the site responsible for all study activities, consulting with the single IRB as early as when first submitting the application may prevent delays in the timeline in the future. Institutions will likely have an internal webpage that details the steps for initiating a multi-site study as well as the documents that are required to fill out as a parent site and for child sites. Setting up a meeting with the single IRB to discuss the order of steps can also serve to ensure all the proper actions are being taken. In the employment of the intervention itself, meetings with the practices to discuss the population’s cultural values and their cultural barriers will help create culturally sensitive intervention materials and improve recruitment and retention rates. Some considerations include scheduling flexibility as some caregivers can only participate in the study in the evenings or weekends, expanding racial categories in demographic questionnaires so that the population is accurately represented and choosing a form of compensation that is accessible to that population (ex. many people in Hawaii shop at CVS).

### Practical implications

Prior work has supported the development and implementation of interventions that are tailored for caregivers and PLWD. Interventions that involve caregivers and PLWD found that educational-focused training correlates to reduced caregiver burden, improved PLWD well-being, and improved caregiver affect [15]. No prior studies have adapted the components of two previously tested dementia care models to develop an intervention to be used by HBPC clinicians. The findings from this study will include the qualitative feedback from caregivers of persons living with dementia and home-based primary care clinicians and practice staff to test the feasibility, acceptability, and fidelity of the Dementia Care Quality at Home intervention. The input from stakeholders and the testing of the intervention will contribute to the steps made toward providing high-quality dementia care in home-based primary care practices. While Dementia Quality of Care at Home adds additional time demands to HBPC staff, feasibility of this model may be increased in Medicare Advantage plans and through the recently released CMS GUIDE model [16].

### Potential challenges

Potential challenges of the study may include difficulties in focus group and open pilot recruitment. Our Hawaii practice has over 800 patients while our Virginia practice has approximately 350 patients. The differences in patient population numbers may lead to different focus group participation rates and perspectives across the two sites. Similarly, both sites have high representations of specific underrepresented communities which may influence the tailoring of the intervention to be most relevant within these specific populations. We see this as a strength of the study since many patients from African American or Black communities (Virginia practice) and AAPI communities (Hawaii practice) are underrepresented in research—particularly those with functional impairment. Additionally, the Hawaii practice has a full care coordination team which may not be reflective of HBPCs on a national level. Phone administration of surveys has the potential of eliciting biased responses but interviews with a research team member who is not a part of the practice may reduce this possibility. Phone-based data collection may be particularly important since there are lower rates of digital access and literacy in this population [1].

### Future directions

If findings demonstrate feasibility and acceptability to both the practices and caregivers of persons living with dementia, the next step will be to plan efficacy and effectiveness studies of the intervention, Dementia Care Quality at Home within additional home-based primary care practices.

## Conclusions

The high prevalence of older adults with dementia who receive home-based primary care and the limited number of dementia-focused interventions in home-based primary care point to the need for more home-based primary care practices that implement high-quality dementia care. This study will adapt a previously successful dementia care model for HBPC practices and caregivers of dementia. By assessing its potential value in homebound persons living with dementia and their caregivers, this study will offer important initial insights into future dementia focused HBPC interventions and help inform the conduct and efficacy of these interventions to support PLWD and their caregivers.

## Data Availability

Data sharing is not applicable to this article as no datasets were generated or analyzed during the current study.

## Abbreviations

PLWD: Persons living with dementia
HBPC: Home-based primary care
IRB: Institutional review board
CGs: Caregivers
EHR: Electronic health record
